# Subjective cognition in schizophrenia and bipolar disorder: Investigation of group differences and associations with objective cognition and clinical characteristics using a novel measure of subjective cognition

**DOI:** 10.1016/j.scog.2025.100345

**Published:** 2025-02-03

**Authors:** Kristoffer Grimstad, Håkon Sørensen, Anja Vaskinn, Christine Mohn, Stine Holmstul Olsen, Ole A. Andreassen, Trine Vik Lagerberg, Ingrid Melle, Merete Glenne Øie, Torill Ueland, Beathe Haatveit

**Affiliations:** a Department of Neurology, Akershus University Hospital, Lørenskog, Norway; b Center for Research and Education in Forensic Psychiatry, Oslo University Hospital, Oslo, Norway; c Centre for Precision Psychiatry, Division of Mental Health and Addiction, Institute of Clinical Medicine, University of Oslo, Oslo, Norway; d National Centre for Suicide Research and Prevention, Institute of Clinical Medicine, University of Oslo, Oslo, Norway; e Section for Clinical Psychosis Research, Division of Mental Health and Addiction, Oslo University Hospital, Oslo, Norway; f Section for Precision Psychiatry, Division of Mental Health and Addiction, Oslo University Hospital, and University of Oslo, Oslo, Norway; g Adult Psychiatry Unit, Division of Mental Health and Addiction, Institute of Clinical Medicine, University of Oslo, Oslo, Norway; h Department of Psychology, University of Oslo, Oslo, Norway; i Department of Research, Innlandet Hospital Trust, Brumunddal, Norway

**Keywords:** Neurocognition, Cognitive impairment, Subjective assessment, Cognitive complaints, Psychotic disorders, Functioning

## Abstract

Cognitive dysfunction is a well-documented feature of schizophrenia (SZ) and bipolar (BD) disorder. The person's subjective experience of cognitive difficulties is less investigated. Here we investigated subjective cognition in SZ and BD compared to healthy controls (HC).

Subjective and objective cognition were assessed in 91 SZ participants, 55 BD participants and 55 HC, applying a novel measure of subjective cognition, the self-assessed cognitive complaints scale (SACCS) and a clinically relevant neuropsychological test battery. The psychometric properties of SACCS were investigated. The relationship between subjective and objective cognition, and subjective cognition and clinical variables were explored in SZ and BD.

The SACCS showed adequate psychometric properties. Clinical groups reported significantly more cognitive complaints than HCs, without differences between SZ and BD. There were no significant associations between subjective and objective cognitive measures. There was a small trend association between subjective cognition and insight in SZ participants, and moderate sized associations between subjective cognition and general psychopathology and functioning in BD participants.

Although SZ participants are more cognitively impaired than BD participants, the two groups report similar levels of subjective cognitive complaints, with no association between subjective and objective cognition. Our results suggest that the expression of subjective cognition is associated with different clinical factors in SZ and BD.

## 1 Introduction

Cognitive dysfunction is a well-documented feature of both schizophrenia spectrum (SZ) and bipolar (BD) spectrum disorders (Bortolato et al., 2015; Schaefer et al., 2013). The neuropsychological profile of SZ is typically characterized by global impairments across cognitive domains (McCutcheon et al., 2023; Schaefer et al., 2013). For BD, there is converging evidence of a global, albeit less severe cognitive dysfunction (Daban et al., 2006; Demmo et al., 2017). Several studies suggest that the cognitive impairments in both disorders are relatively independent of clinical symptoms, and tend to persist when psychotic, manic or depressive symptoms are in remission (Bora and Özerdem, 2017; Demmo et al., 2017; Tabarés-Seisdedos et al., 2008; Van Rheenen et al., 2020).

An important but less investigated phenomenon is the person's subjective experience of cognitive difficulties. Some have argued that subjective cognition in SZ is as clinically relevant as objective cognition given that the former is an expression of self-perceived difficulties in real-life situations not captured by objective testing alone (Chang et al., 2015; Tong et al., 2019). From a clinical perspective, neglecting subjective cognitive complaints in favor of objective test results may diminish patients' motivation for treatment (Treichler et al., 2019). Whereas objective cognitive functioning is assessed by neuropsychological testing, subjective functioning is assessed by interview or self-report. In SZ commonly used validated instruments of subjective cognition include the Measure of Insight into Cognition – Self Report (MiC-SR) (Medalia et al., 2008), the Schizophrenia Cognition Rating Scale (SCoRS) (Keefe et al., 2006), and the Subjective Scale to Investigate Cognition in Schizophrenia (SSTICS) (Stip et al., 2003). In BD, the Cognitive Complaints in Bipolar Disorder Rating Assessment (COBRA) (Rosa et al., 2013) is a frequently used scale.

Participants with SZ and BD consistently report more subjective cognitive complaints compared to healthy controls (Lin et al., 2019; Luo et al., 2020; Potvin et al., 2014; Rosa et al., 2013; Svendsen et al., 2012), with some notable exceptions. For instance, Chan et al. (2008) found that SZ participants did not report disproportionately higher subjective complaints than controls, and Tatay-Manteiga et al. (2019) found no differences in subjective cognition between BD participants and controls.

The association between subjective and objective cognitive functioning in SZ has been explored in several studies, with inconclusive findings (Homayoun et al., 2011). A meta-analysis by Potvin et al. (2014) found a small association between subjective and objective cognition, while others have found associations in a subgroup of SZ participants (Raffard et al., 2020) or in specific cognitive domains (Baliga et al., 2020). Findings in BD are similar, some report associations between subjective and objective cognitive function (Demant et al., 2015; Lin et al., 2019; Rosa et al., 2013), while others do not (Burdick et al., 2005; Harvey et al., 2015; Svendsen et al., 2012; van der Werf-Eldering et al., 2011). In sum, the association between subjective and objective cognition in SZ and BD is complex and equivocal.

The same holds true for the association between subjective cognition and clinical factors. The meta-analysis by Potvin et al. (2014) found no correlations between subjective cognition and positive or negative symptoms in SZ. However, they found correlations between subjective complaints and insight into illness, as well as with depressive symptoms. The association between cognition and depressive symptoms has later been established/confirmed in several other studies in SZ (Burton et al., 2016; Chang et al., 2015; Durand et al., 2015; Prouteau et al., 2017; Raffard et al., 2020; Shin et al., 2016; Tong et al., 2019; Treichler et al., 2019). Regarding BD, the majority of recent studies have found associations between subjective cognitive complaints and affective symptoms (Demant et al., 2015; Harvey et al., 2015; Lin et al., 2019; Luo et al., 2020; Miskowiak et al., 2012; Svendsen et al., 2012; van der Werf-Eldering et al., 2011).

Despite several similarities in the SZ and BD literature pertaining to subjective cognition, to our knowledge, no previous studies have directly compared the two groups. A clinically developed measure of subjective cognition (by authors TU and MØ) has been implemented in the research protocol at our Centre and has been completed by these two groups. Thus, we now have the opportunity to investigate subjective and objective cognition in SZ and BD participants. We also have data from healthy controls (HC). More specifically we investigate:

1. the psychometric properties of a novel measure of subjective cognition, the self-assessed cognitive complaints scale (SACCS), including the reliability and the validity of the scale.
2. whether there are differences in subjective and objective cognition between SZ, BD and HC.
3. whether there are associations between subjective and objective cognition in SZ and BD.
4. whether there are associations between subjective cognition and clinical characteristics in SZ and BD.

## 2 Methods

### 2.1 Participants

This study included 91 participants with schizophrenia spectrum disorders (SZ), including diagnosis of schizophrenia (59 %), schizophreniform disorder (10 %), schizoaffective disorder (9 %), and psychosis not otherwise specified (NOS, 22 %), 55 participants with bipolar disorder spectrum (BD), including diagnoses of bipolar I (60 %), bipolar II (31 %), and bipolar NOS (9 %), and 55 healthy controls (HC). The majority of participants in the clinical groups were using antipsychotic and/or mood-stabilizing medications (SZ group: 93,4 %, BD group: 76,4 %). In the SZ group, 3,3 % of individuals were prescribed anxiolytics or hypnotics, compared to 14,5 % in the BD group. All participants were recruited through the ongoing Thematically Organized Psychosis (TOP) study at the Oslo University Hospital. The clinical sample was recruited from in- and outpatient psychiatric clinics at four major hospitals in the Oslo region. HCs were randomly selected from the same catchment area and invited to participate via letters. The exclusion criteria for all study participants were a medical history of severe head trauma, any neurological disorder, and mental retardation (defined as IQ < 70). Participants were required to have an acceptable level of comprehension of the Norwegian language. HCs were excluded if they or any close relative had a lifetime history of severe mental disorder, or any substance abuse in the last year. All participants gave written consent, and the study was approved by the Regional Committee for Medical Research Ethics (REK), and the Norwegian Data Protection Authority, and conducted in accordance with the Helsinki declaration.

### 2.2 Assessment of clinical characteristics

Trained clinical psychologists or physicians carried out clinical assessments. Diagnostic assessments were done using the Structured Clinical Interview for DSM-IV Axis I disorders, SCID-I (First et al., 1997). Depressive symptoms were assessed using the Calgary Depression Scale for Schizophrenia (CDSS) (Addington et al., 1990) and psychotic symptoms using the Positive and Negative Syndrome Scale (PANSS) (Kay et al., 1987). PANSS scores were calculated using Wallwork's five-factor model (Wallwork et al., 2012). Clinical insight was measured with item G12 in the PANSS. For the BD group current manic symptoms were assessed using the Young Mania Rating Scale (YMRS) (Young et al., 1978). Global level of functioning was assessed using the split version of the Global Assessment of Functioning Scale with separate scores for symptoms (GAF—S) and a functioning (GAF—F) (Pedersen et al., 2007). Participants were assessed during a stable phase of illness and completed neuropsychological assessment within two weeks after their clinical evaluation.

### 2.3 Assessment of objective cognition

Neuropsychological assessments were performed by psychologists/masters in neuroscience (patients) or psychology students (HC) trained in standardized neuropsychological assessment. Objective cognition was assessed using the MATRICS Consensus Cognitive Battery (MCCB) (Nuechterlein et al., 2008) excluding the social cognitive domain. Raw scores were converted to demographically corrected T-scores based on published norms for the tests. A global cognitive composite score was calculated using the mean of T-scores from the nine MCCB subtests. IQ-scores were obtained using Matrix Reasoning and Vocabulary from the Wechsler Abbreviated Scale of Intelligence (WASI) (Wechsler, 1999).

### 2.4 Assessment of subjective cognition

Subjective cognition was assessed using the SACCS. The scale was originally developed by Ueland, Øie and Tandberg for clinical use to acquire qualitative knowledge concerning subjective cognitive impairment (personal communication). It consists of 18 items with a Likert type scale that asks subjects to rate frequency and/or severity of common cognitive problems on a scale ranging from “0-never” to “4- very often”. E.g: “do you find it difficult to keep focused over time?” The self-report form was designed both to assess the cognitive domains typically impaired in clinical samples and to be closely linked to the objective measurement of cognitive function. In order to match and compare subjective and objective cognition the 18 items were split into four categories mirroring cognitive domains typically assessed by neuropsychological test batteries: attention, learning and memory, processing speed and executive function.

### 2.5 Statistical analyses

Statistical analyses were conducted by using IBM SPSS Statistics version 26 (IBM Corp., 2019). For demographical data, Chi-square tests were applied when comparing groups on categorical data, and student *t*-tests and analysis of variance (ANOVA) were used when investigating group differences on continuous data.

The psychometric properties of the SACCS were investigated to address questions related to reliability and validity of the scale. The frequency of the total scale score and the subscale scores were first visually inspected. We then checked the total scale score for normality using the Kolmogorov–Smirnov test. Next, we investigated the internal consistency of the scale reporting Cronbach's α for the subscales and for the total scale, based on the total sample. Internal consistency was examined using Pearson correlations analyses *(r*) both between the sub scale scores and the full-scale score and between the four subscale scores.

To examine differences in subjective and objective cognition between the two clinical groups and HC we used ANOVA. The SACCS total and domain scores, as well as objective cognition domains and composite scores, were entered as dependent variables and group (SZ/BD/HC) was entered as the between variable. In the case of a significant effect of group, follow-up analyses of variance were undertaken, with potential confounding variables included (ANCOVA). Confounding variables were those demographic variables that differed significantly between groups. We chose not to include education and IQ, as these variables would remove inherent characteristics of the clinical groups, and therefore opted to include only gender and age (see Table 1). We chose Bonferroni post-hoc test to control for multiple comparisons, where pairwise comparisons of groups were conducted to identify specific group differences.

**Table 1:** Demographic and clinical characteristics.

	SZ (*n* = 91)	BD (*n* = 55)	HC (n = 55)	Statistics	p	Post hoc analysis
DEMOGRAPHICS						
Gender, male, n (%)	58 (63.7)	23 (41.8)	33 (60.00)	*X*^*2*^(2) = 7.04	0.03	
Age, years, M (range)	28.92 (18–59)	32.78 (18–63)	35.94 (17–73)	*F*(2) = 7.53	0.001	SZ < HC
Education, years, M (range)	12.37 (9–18)	13.55 (9–17)	14.93 (9–19)	*F*(2) = 22.02	<0.001	HC > SZ, BD; BD > SZ
WASI IQ, M (SD)	101.49 (12.49)	111.12 (11.58)	116.4 (9.85)	*F*(2) = 29.70	<0.001	SZ < BD, HC
CLINICAL CHARACTERISTISCS						
PANSS positive, M (SD)	8.43 (3.15)	5.14 (1.53)		*t*(138,68) = 8.43	<0.001	
PANSS negative, M (SD)	13.84 (5.52)	8.98 (4.04)		*t*(136,68) = 6.08	<0.001	
PANSS disorganized, M (SD)	5.51 (2.39)	3.96 (1.31)		*t*(142.96) = 5.04	<0.001	
PANSS excitement, M (SD)	5.28 (1.59)	4.94 (1.33)		*t*(144) = 1.32	0.187	
PANSS depression, M (SD)	7.47 (2.68)	7.98 (2.87)		*t*(144) = − 1.08	0.281	
PANSS G12 insight, M (SD)	2.57 (1.26)	1.56 (0.83)		*t*(143) = 5.79	<0.001	
CDSS, M (SD)	4.42 (4.11)	5.53 (4.62)		*t*(144) = −1.50	0.134	
YMRS, M (SD)		2.02 (2.99)				
GAF-S, M (SD)	49.13 (12.19)	62.76 (9.37)		*t*(144) = −7.11	<0.001	
GAF-F, M (SD)	48.53 (11.35)	62.44 (11.28)		*t*(143) = −7.15	<0.001	

Note: WASI – Wechslers Abbreviated Scale of Intelligence, PANSS – Positive and Negative Syndrome Scale, CDSS – Calgery Depression Scale for Schizophrenia, YMRS – Young Mania Rating Scale, GAF-S – Global Assessment of Function - Symptoms, GAF-F – Global Assessment of Function - Function.

Associations between objective and subjective cognition and between subjective cognition and clinical characteristics were analyzed using Pearson's correlation analyses in the two clinical groups separately.

All effects were corrected for multiple testing using the Bonferroni method (α = 0.05/x tests). For all post hoc tests the statistical threshold was set to a nominal *p* ≤ .05. Cases with missing data were listwise/pairwise deleted in all analyses. In our sample a total of 77 participants in the SZ group, 45 participants in the BD group, and 53 participants in the HC group completed a full neuropsychological assessment.

## 3 Results

### 3.1 Demographic and clinical characteristics

Demographic and clinical characteristics are presented in Table 1. The BD group consisted of significantly fewer males compared to the SZ and HC groups. Participants in the HC group were significantly older than participants in the SZ group. There were significant differences in education between all groups, of which the HC group had the longest education and the SZ group the shortest. Additionally, the SZ group had significantly lower IQ compared to the BD and HC groups. Furthermore, the SZ group had significantly higher scores on all clinical measures compared to the BD group, except for excitement and depression symptoms assessed using PANSS, and depressive symptoms assessed using CDSS, where there were no significant differences between the groups.

### 3.2 Psychometric properties of the SACCS

Reliability data is presented in Table 3. Visual inspection of the subscale and full-scale data showed acceptable range of scores. The One-sample Kolmogorov-Smirnoff Test yielded non-significant result for the full scale (*p* = .20), indicating normality of the data. The full scale, and Attention, Processing speed and Executive function subscales of the SACCS all had high reliabilities, with Cronbachs *α* ranging from 0.75 to 0.92. Learning and memory subscale had a somewhat lower reliability, with a Cronbachs *α* = 0.69. Mean inter-item correlations varied between *r* = 0.42 to *r* = 0.57 within the four subscales, and *r* = 0.46 for the full scale. The mean item-total correlations ranged from *r* = 0.50 to *r* = 0.69. Bivariate correlations between mean full scale and subscale scores ranged from *r* = 0.85 to *r* = 0.91, while correlations between mean subscale scores ranged from *r* = 0.70 to *r* = 0.77.

### 3.3 Objective cognition

**Fig. 1:**
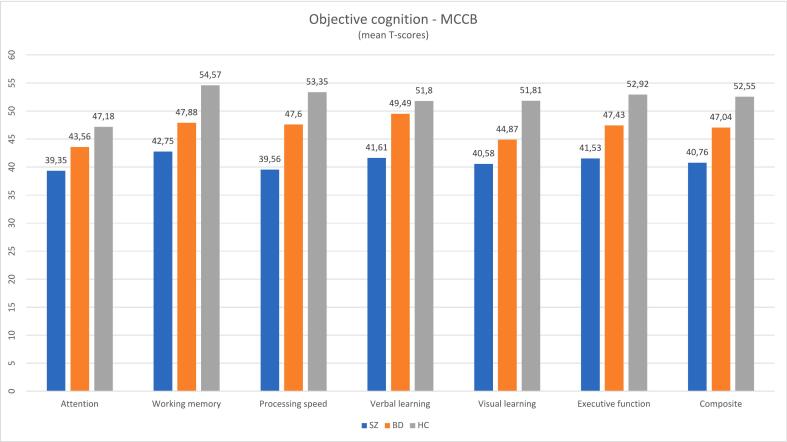
Objective cognition.

Table 2 and Fig. 1 illustrate objective cognitive performance across cognitive domains in participants with SZ, BD and in HC. The SZ group obtained significantly lower scores in all cognitive domains compared to the HC group. The BD group obtained significantly lower scores in the domains of processing speed, working memory, visual learning, executive function, and global composite, compared to the HC group. Compared to the BD group, the SZ group obtained significantly lower scores in all domains, except in visual learning.

**Table 2:** Objective and subjective cognition.

	SZ	BD	HC	Statistics	p	Post hoc analysis
OBJECTIVE COGNITION (MCCB)						
Attention, M (SD)	39.35 (10.12)	43.56 (7.93)	47.18 (7.85)	*F*(2) = 12.44	<0.001	SZ < HC, BD
Working memory, M (SD)	42.75 (10.43)	47.88 (7.77)	54.57 (7.22)	*F*(2) = 27.76	<0.001	SZ < HC, BD; BD < HC
Processing speed, M (SD)	39.56 (7.97)	47.60 (9.07)	53.35 (7.71)	*F*(2) = 46.94	<0.001	SZ < HC, BD; BD < HC
Verbal learning, M (SD)	41.61 (11.48)	49.49 (9.08)	51.80 (11.34)	*F*(2) = 16.08	<0.001	SZ < HC, BD
Visual learning, M (SD)	40.58 (10.91)	44.87 (9.86)	51.81 (9.73)	*F*(2) = 19.01	<0.001	HC > SZ, BD
Executive function, M (SD)	41.53 (10.24)	47.43 (10.60)	52.92 (8.62)	*F*(2) = 21.36	<0.001	SZ < HC, BD; BD < HC
Composite, M (SD)	40.76 (7.05)	47.04 (6.67)	52.55 (5.71)	*F*(2) = 52.36	<0.001	SZ < HC, BD; BD < HC
SUBJECTIVE COGNITION (SACCS)						
Attention, M (SD)	11.73 (3.89)	11.93 (4.10)	6.95 (2.89)	*F*(2) = 34.20	<0.001	HC < SZ, BD
Learning and memory, M (SD)	4.92 (2.22)	5.00 (2.15)	2.55 (1.65)	*F*(2) = 27.22	<0.001	HC < SZ, BD
Processing speed, M (SD)	4.88 (2.42)	4.56 (2.47)	1.98 (1.52)	*F*(2) = 31.36	<0.001	HC < SZ, BD
Executive function, M (SD)	12.11 (4.64)	12.82 (4.97)	5.62 (3.49)	*F*(2) = 46.33	<0.001	HC < SZ, BD
Total score, M (SD)	33.64 (11.16)	34.31 (12.10)	17.09 (8.38)	*F*(2) = 48.77	<0.001	HC < SZ, BD

**Table 3:** Reliability measures for the SACCS.

	Full scale	Scale 1 Attention	Scale 2 Learning and memory	Scale 3 Processing speed	Scale 4 Executive function
Number of items (n)	18	5	3	3	7
Mean item-total correlation (r)	0.660	0.696	0.506	0.578	0.642
Mean inter-item correlation (r)	0.465	0.572	0.424	0.499	0.486
Cronbach's alpha	0.940	0.870	0.689	0.750	0.868

Results are reported for total sample (*n* = 201). SACCS – Self-assessed cognitive complaints.

**Table 4:** Associations between subjective cognition (SACCS total score), objective cognition and clinical characteristics.

	SZ	BD
Objective cognition composite	*r* = −0.048, *p* = .672	*r* = −0.013, *p* = .934
PANSS positive	*r* = 0.155, *p* = .143	*r* = 0.090, *p* = .512
PANSS negative	*r* = −0.083, *p* = .433	*r* = 0.129, *p* = .353
PANSS disorganized	*r* = 0.010, *p* = .925	*r* = 0.286, *p* = .034⁎
PANSS excitement	*r* = −0.017, *p* = .876	*r* = 0.102, *p* = .460
PANSS depression	*r* = 0.190, *p* = .071	*r* = 0.357, *p* = .007⁎
PANSS G12 insight	*r* = −0.264, *p* = .011⁎	*r* = 0.153, *p* = .264
CDSS	*r* = 0.188, *p* = .074	*r* = 0.351, *p* = .009⁎
YMRS		*r = 0.285, p = .039*⁎
GAF-S	*r* = −0.090, *p* = .396	*r* = −0.451, *p* = .001⁎⁎
GAF-F	*r* = −0.052, *p* = .625	*r* = −0.437, *p* = .001⁎⁎

⁎ Significant at the 0.05 level.

⁎⁎ Significant after Bonferroni correction (*a* = 0.0045).

### 3.4 Subjective cognition

**Fig. 2:**
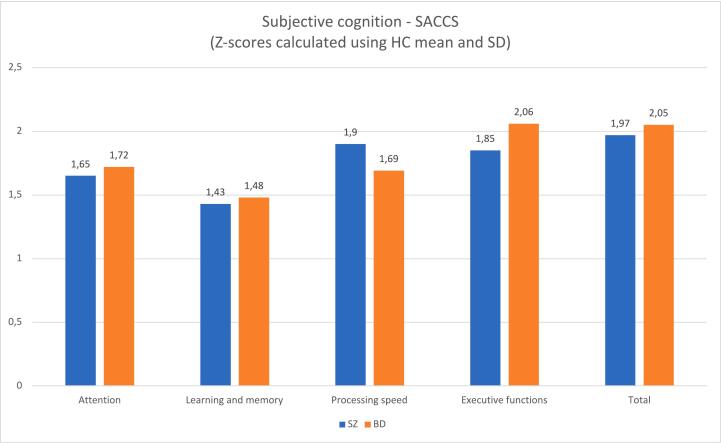
Subjective cognition.

Subjective cognition in the SZ, BD and HC groups are presented in Table 2 and illustrated in Fig. 2. The HC group reported significantly fewer cognitive complaints in all domains, including Attention (*F*_*(2)*_ = 34.2, *p* < .001), Learning and memory (*F*_*(2)*_ = 27.2, *p* < .001), Processing speed (*F*_*(2)*_ = 31.4, *p* < .001), Executive function (*F*_*(2)*_ = 46.3, *p* < .001), and on the SACCS total score (*F*_*(2)*_ = 48.8, *p* < .001) compared to the clinical groups. The clinical groups reported a similar degree of cognitive complaints, and the difference between groups did not reach statistical significance in any domains. Controlling for potential confounders (age and sex) did not affect the results (all *p* values > .05).

### 3.5 Associations between subjective cognition, objective cognition and clinical characteristics

There was no significant correlation between the SACCS total score and the cognitive composite score in the SZ group (*r* = −0.048, *p* = .672), nor in the BD group (*r* = −0.013, *p* = .934). In the SZ group, a small and significant correlation was found between the SACCS total score and clinical insight, measured with item G12 in the PANSS (*r* = −0.264, *p* = .011). However, after correcting for multiple testing, the effect was no longer statistically significant. For the BD group, significant and moderate sized correlations were found between the SACCS total score and several clinical variables, including general psychopathology (GAF-S), depressive symptoms (CDSS, PANSS depression), disorganized symptoms (PANSS disorganized), manic symptoms (YMRS), and functioning (GAF—F). After correcting for multiple testing only correlations between GAF-S and GAF-F function remained statistically significant (see Table 4).

## 4 Discussion

The current study showed that our measure of subjective cognitive complaints, the SACCS, has adequate psychometric properties. Both SZ and BD participants report significantly more subjective cognitive complaints than HC. The SACCS showed weak concurrent validity with objective cognition as evidenced by the small and non-significant correlation with the cognitive composite score. We argue that the SACCS has satisfactory face validity and can be used to estimate participants' level of subjective cognitive complaints.

We found no significant difference in subjective cognitive complaints between the SZ and BD participants. Both groups reported an equal degree of subjective cognitive complaints, in terms of specific cognitive domains and in the overall total score. This finding is somewhat unexpected, given that the SZ group performed significantly worse than the BD group on most objective cognitive measures, including attention, working memory, processing speed, verbal learning, executive function, and global cognition (composite).

There were significant differences between the groups in age and gender. However, when covarying for age and gender this did not alter the original findings of no difference between the clinical groups in subjective cognitive complaints. There were also differences between the groups in IQ and education. We have however chosen not to covary for these factors. The rationale for this is that individuals with SZ are often intellectual compromised (Parellada et al., 2017; Woodberry et al., 2008), and controlling for IQ may, in a sense, remove the effect for the illness itself. A similar argument can be made for education. The onset of illness in both SZ and BD often occurs early in adulthood, frequently disrupting educational trajectories. Differences between HC and clinical groups may, therefore reflect illness severity rather than cognitive functioning.

We found no association between subjective cognitive complaints and objective cognition in either of the clinical groups. This is consistent with the majority of previous findings both in SZ (Potvin et al., 2014) and BD (Burdick et al., 2005; Harvey et al., 2015; Schouws et al., 2012; Svendsen et al., 2012; van der Werf-Eldering et al., 2011), although some studies find an association (Baliga et al., 2020; Demant et al., 2015; Lin et al., 2019; Raffard et al., 2020; Rosa et al., 2013). Burton et al. (2016) suggested that a substantial portion of individuals with SZ, despite having objective cognitive impairments, appear unaware of their deficits. Such lack of awareness could potentially explain why we did not find an association in the SZ group. Similarly, a potential overestimation of subjective cognitive dysfunction could explain the lack of relationship in BD.

Like previous studies, we found different clinical characteristics associated with subjective cognition in the two diagnostic groups. In the SZ group a lower degree of insight into illness was associated with fewer reported cognitive complaints. While the association was not statistically significant after correcting for multiple testing, the finding is in line with the meta-analysis by Potvin et al. (2014) and a recent study by Chuang et al. (2019). This is interesting from a clinical perspective, as many individuals suffering from psychotic symptoms will often reject the notion of being mentally ill as an explanation for their experiences, a condition known as anosognosia. Our trend level findings may indicate that insight into illness in SZ not only applies to psychotic symptoms, but also influences the individual's experience of cognitive difficulties.

Our results show that subjective cognitive complaints were not associated with psychotic symptoms, depressive symptoms, general symptoms of psychopathology, or functioning in SZ. Previous studies have consistently found an association between subjective cognition and depressive symptoms in SZ (Burton et al., 2016; Chang et al., 2015; Durand et al., 2015; Potvin et al., 2014; Prouteau et al., 2017; Raffard et al., 2020; Shin et al., 2016; Tong et al., 2019; Treichler et al., 2019). It is well known that depressed individuals tend to overestimate their cognitive impairment (Schwert et al., 2018). The lack of significant association in the current study may be related to low levels of depressive symptoms in our SZ sample or to the small sample size.

In the BD group, the degree of subjective cognitive complaints was significantly associated with low levels of functioning and higher levels of general psychopathology. There were also trend level associations between subjective cognitive complaints and symptoms of disorganization, depression, and mania. These results are partly in line with the majority of studies that show associations between subjective cognitive complaints and affective symptoms (Demant et al., 2015; Harvey et al., 2015; Lin et al., 2019; Luo et al., 2020; Miskowiak et al., 2012; Svendsen et al., 2012; van der Werf-Eldering et al., 2011). Rosa et al. (2013) highlights the potential negative impact of depressive symptoms on subjective cognition, given that depressed individuals tend to overestimate their deficits. Similarly, Demant et al. (2015) suggests that a depression bias is in effect, in which depression severity may impact individuals` self-rating of cognitive function. Following this line of reasoning, Harvey et al. (2015) proposed that BD participants may tend to index their cognitive functioning by their distress level.

The link between global functioning and subjective cognition is less explored. However, Demant et al. (2015) and Luo et al. (2020) found an association between subjective cognition and global functioning among BD participants. Since our study is cross-sectional, we could not explore potential causal relationships between subjective cognition and global functioning. However, one possible explanation may be that individuals with a high degree of subjective cognitive complaints also have a high general symptom burden, which in turn affects their capacity to manage everyday activities, such as work or education.

Most participants in the clinical groups were using antipsychotic and/or mood-stabilizing medications. Studies have shown mixed effects with regard to the impact of these medications on cognition in SZ (Feber et al., 2025) and BD (Xu et al., 2020) but generally indicate minor effects when taken at optimal doses. Unfortunately, in the current study we did not have detailed information about medication type or dosage, which prevented us from controlling for medications in the analyses. Thus, although we cannot rule out that medications could have some impact, we do not think they can explain the entirety of the results.

Taken together, our findings reveal significant group differences between SZ and BD participants regarding which clinical factors are associated with subjective cognitive complaints. These findings can possibly contribute to explaining the lack of group difference in subjective cognition. Although we have not investigated causal relationships, a low level of insight into illness may underlie fewer subjective cognitive complaints reported in SZ group. Conversely, high general symptom burden and low level of functioning, may affect BD group in reporting more subjective cognitive complaints. The sum of these factors may collectively contribute to the similarity in the levels of subjective cognitive complaints reported by the two clinical groups.

The current study has several strengths. To the best of our knowledge, no previous studies have investigated the association between subjective and objective cognition in both SZ and BD participants, as well as including a HC group. There are also some limitations that warrant mentioning. One limitation, is the use of a measure of subjective cognition that has not been validated in an independent sample. Unfortunately, as other measures of subjective cognition had not been translated into Norwegian at the start of our study, we did not have the opportunity to compare the SACCS to other instruments measuring subjective cognition. Thus, we were unable to investigate the SACCS convergent validity. Since assessments were conducted at a single time point, we were unable to evaluate the SACCS test-retest reliability. It could also be argued that we have too few measures of symptom severity, which weakens assumptions related to the SACCS divergent validity.

Future studies should address the limitations mentioned above. Additionally, it would be valuable for future research to control for potential effects of medication, covary for insight and other clinical variables, as well as to include a larger sample size with participants with more severe symptomatology.

In sum our results suggest that the SACCS has adequate psychometric properties. SZ and BD participants report significantly more subjective cognitive complaints than HC. The two clinical groups report similar levels of subjective cognitive complaints, despite significant differences in objective cognition. No associations were found between objective and subjective cognition, and our findings suggest that subjective cognitive complaints are associated with different clinical factors in SZ and BD. The current findings suggest that the subjective level of cognition is relevant for treatment and care of SZ and BD in the clinic.

## CRediT authorship contribution statement

**Kristoffer Grimstad:** Writing – original draft, Methodology, Formal analysis, Conceptualization. **Håkon Sørensen:** Writing – original draft, Methodology, Formal analysis, Conceptualization. **Anja Vaskinn:** Writing – review & editing. **Christine Mohn:** Writing – review & editing. **Stine Holmstul Olsen:** Writing – review & editing. **Ole A. Andreassen:** Writing – review & editing, Funding acquisition. **Trine Vik Lagerberg:** Writing – review & editing. **Ingrid Melle:** Writing – review & editing, Funding acquisition. **Merete Glenne Øie:** Writing – review & editing. **Torill Ueland:** Writing – review & editing, Supervision, Conceptualization. **Beathe Haatveit:** Writing – review & editing, Supervision, Conceptualization.

## Funding information

This study was funded by the 10.13039/501100005416Research Council of Norway (grant number 223273). The funding source had no involvement in the study design or preparation of the article.

## Declaration of competing interest

Ole A. Andreassen is a consultant to Cortechs.ai and Precision Health AS, and has received speaker's honorarium from Lundbeck, Sunovion, Janssen and Otsuka. Other authors report no conflicts of interests.
